# Dignity restored: the power of treatment first

**DOI:** 10.1017/S109285292400052X

**Published:** 2024-12-23

**Authors:** Jhilam Biswas

**Affiliations:** Department of Psychiatry, Mass General Hospital and Brigham and Women’s Hospital, Boston, MA, USA

**Keywords:** health equity, anosognosia, global mental health, antipsychotic medication, healthcare burnout, Rogers Guardianship, substituted judgment, psychiatric commitment

## Abstract

The perspective article explores systemic issues in psychiatric care, particularly the barriers to timely treatment and the ethical dilemmas involved in involuntary interventions. It further examines the impact of anosognosia—lack of disease insight—on treatment, noting the difficulties in managing care for those unaware of their illness, and scrutinizes training materials from international organizations that might mislabel necessary psychiatric practices as human rights violations, thereby complicating the care landscape. The discussion extends to the legal and societal implications of psychiatric interventions, using Massachusetts’ Rogers Guardianship as a case study to highlight the consequences of legalistic approaches to mental health treatment.

The article calls for destigmatizing psychiatric treatment and integrating robust, evidence-based practices to improve patient outcomes and healthcare equity. The global mental health policy landscape is urged to recognize the critical role of psychiatric care in restoring health and dignity to individuals with serious mental illnesses, advocating for a more nuanced understanding and application of human rights in mental health.

“I couldn’t tell where my body ended and the world began. Voices were speaking to me from the television, telling me I was doomed.”

In “*The Quiet Room: A Journey Out of the Torment of Madness,”* Lori Schiller, who lives with a diagnosis of Schizoaffective Disorder, vividly recounts her intense struggles with psychosis. Schiller’s narrative provides profound insight into the unsettling and often terrifying world caused by this condition, characterized by feelings of isolation and being inundated with fear and distressing delusions.^1^ Psychosis, far from harmless, carries a significant burden of trauma, evidenced by the tragically high suicide rates among individuals diagnosed with diseases associated with psychosis. The battle with intrusive perceptual disturbances and paranoia presents a relentless test of human endurance, and this painful state requires alleviation through many modalities and, importantly, medical treatment.

The symptom of anosognosia, or lack of insight and awareness of the disease, can make it challenging to provide care as the patient is not aware they are ill. This symptom is a common symptom of schizophrenia, and this lack of insight often results in stopping and starting medications, frequent hospitalizations, chronic illness, and increasing risk of homelessness and incarceration.^2^ Without a thorough and data-driven assessment of the patient, psychiatric providers may misdiagnose anosognosia symptoms as an ambivalence to care. Some researchers think that the psychoanalytic ideas of denial and defense mechanisms, which historically have played a prominent role in psychiatric education, may make it harder for clinicians to spot anosognosia correctly^3^ as it could be misconstrued as defensiveness. Beyond clinical observation, recent brain imaging studies linking cognitive awareness problems in the neurocircuitry of the brain to mental illness,^4^ ^,^^5^ as well as blood gene expression biomarkers,^6^ have also led to supporting evidence of anosognosia in schizophrenia. These studies are explained in more detail elsewhere in this Special Issue.

This article explores the challenges of delivering timely psychiatric treatment and its vital role in restoring health and dignity in individuals with serious mental illness. The piece examines potential pitfalls in international organizations’ training materials that label certain psychiatric interventions as human rights violations. A case study from a state in the United States demonstrates how rights-based legal models in psychiatric care that adhere to similar ideas illustrated in the international training materials have increased access barriers to care and increased adverse events to both patients and healthcare staff.

The article discusses how compulsory yet necessary psychiatric interventions when used appropriately and administered respectfully for patients with severe illnesses who have been determined to lack decision-making capacity, can improve well-being, restore a patient’s dignity, and reduce caregiver fatigue and demoralization.^7^ Failure to provide necessary psychiatric interventions due to stigma around psychiatric treatment raises serious ethical concerns about healthcare access, equity, and delivery.^8^

## The global mental health policy landscape and the stance on intensive psychiatric interventions

Global perspectives on psychiatric care and treatment vary widely as many countries continue to navigate the complex socioeconomic challenges of providing care in varying legal contexts.^9^ Although standardization is difficult to achieve globally, the United Nations Convention on the Rights of Persons with Disabilities (CRPD) and the World Health Organization (WHO) initiatives have articulated policies to integrate important human rights principles into global mental health. The organizations have advocated for equal and non-discriminatory practices, rights to informed consent, the right to rehabilitation and recovery, access to justice, the right to privacy, and participation in public life.^10^ More recently, organizational efforts have led to the development of training modules that are aimed at reducing human rights violations in treating individuals with mental illnesses.

In 2019, the World Health Organization (WHO) QualityRights Initiative released training materials to guide improved mental health care.^11^ These training modules cover several topics, such as patient-centered recovery, supportive communication, and de-escalation techniques, to reduce abuse and neglect in mental health practices. The training modules have many positive aspects and aim to reduce a variety of abuses that have been documented to occur in low-resourced settings that are attempting to provide mental health care.^12^ However, the training module content does critique acute and necessary psychiatric interventions. The modules portray psychiatric providers and psychopharmacology in a stigmatizing manner by equating medically necessary interventions—which physicians and other providers use as part of evidence-based practice to restore health and well-being—with violence and abuse.

The initiative includes five core training modules focused on human rights, mental health, and human rights, legal capacity and the right to decide, recovery, and the right to health, as well as freedom from coercion, violence, and abuse. Specifically, in the materials for “Freedom from coercion, violence, and abuse,” the training explicitly states that practices such as using involuntary medications in the inpatient unit, forced admissions to psychiatric units, and forced treatments in the community constitute “examples of violence and coercion.”

While these training modules incorporate crucial safeguards to improve mental healthcare, they also introduce a paradox. The challenge lies in reconciling the QualityRights Initiative’s advocacy for rehabilitation and recovery with its simultaneous classification of potentially necessary interventions, like brief involuntary treatments for recovery, as violations. This dichotomy highlights the ongoing debate within mental health care about balancing protective measures for autonomy with the clinical realities faced by medical professionals and caregivers in treating serious mental illness.^13^

In an editorial in the British Journal of Psychiatry, two physicians describe the negative portrayal of psychiatry and psychopharmacology in the WHO QualityRights training module, stating:

Psychiatrists are mentioned at least 16 times, 13 of those references are negative. Practitioners are portrayed as unsympathetic, dismissive, and heavy-handed in prescribing medication. This is stigmatizing of the profession and could create a further barrier to individuals accessing healthcare.

Similarly, psychotropic medication is represented in a highly negative light. There are multiple references to the adverse effects of medication; at least 14 references to medication were identified, none of which mentioned the advantages of pharmacological interventions. Psychotropics have the potential to dramatically improve the quality of an individual’s life. Although they are associated with both risks and benefits, this is true of all medications. Their depiction in the training materials does not reflect their robust evidence base.^14^

Classifying essential psychiatric interventions in acute care settings as human rights violations is highly problematic. While global instances of misuse certainly need addressing, such classifications may impact health systems making evidence-based decisions and good-faith efforts to provide treatment. Medications are an essential part of the recovery from psychosis, mania, and other debilitating symptoms of mental illness. Stigmatizing these essential treatments and how they are administered as human rights violations risks undermining their legitimacy and effectiveness at restoring a person’s dignity, right to healthcare, and freedom from a distressing emotional state.

There is a significant gap in international policy-making discussions concerning biological disease processes and the management of agitated psychosis and other severe conditions when they become unpredictable, dangerous, and resistant to treatment. This is particularly notable in the context of policies that criticize the use of involuntary neuroleptic treatment, and the ethics of its use require multilayered conversations with all stakeholders.^15^ ^,^^16^

One example of the excessive use of adversarial legal frameworks to protect the right to refuse treatment for severe and acute mental illness in one state in the United States is called the Rogers Guardianship Hearing. This court hearing, used in Massachusetts, adheres to the substituted judgment principles. The case law that led to this procedure and its aftermath illustrate the potential stigmatization of medication and the delays to treatment that can arise from solely relying on legal mechanisms to dictate already scarce medical care.

## The Massachusetts experiment: the rogers case decision and its impact

In 1970, the Massachusetts legislature established that admission to a psychiatric hospital did not necessarily equate to incompetence around treatment. This decision set the stage for the legal and ethical stance that a formal assessment of incompetence in medical decision-making is required before treatment can be administered against a patient’s will to protect the patient’s right to refuse medications. Massachusetts ended up with a complex process called the Rogers Guardianship, resulting from a series of legal battles that began in 1975 and culminated in a series of district, state, and Supreme Court decisions over eight years. Starting with the case named Rogers v. Okin, the final decision and following precedent were made in 1983 in a district court in the case called Rogers v. Commissioner,^17^ Rogers v. Okin,^18^ Mills v. Rogers,^19^ Rogers v. Commission of the Department of Mental Health.^20^

This case became an important precedent and, in forensic psychiatry literature, represented the rights-driven model in state case law for psychiatric care. Notably, the Rogers case highlighted employing the substituted judgment approach over a best-interests model for decisions on necessary treatment for individuals suffering acute mental illness. This method asks a judge, rather than a doctor, to discern whether the patient, deemed incompetent, would agree to take antipsychotic medication if they could make medical decisions for themselves. This exercise introduced a paradoxical dilemma in psychiatric contexts: if incompetence is due to a mental condition that improves with medication, making the patient competent, then theoretically, the now-competent individual would not require the medication. Taken to its logical extreme, this would suggest that involuntary treatment could never be justified, irrespective of the apparent need.^21^ ^,^^22^

However, case law decisions made at the time of the Rogers ruling did not all reduce physicians’ expertise to clinical testimony in scenarios involving compulsory treatment. The following cases represented case law in the treatment-driven model. In the 1982 case of Youngberg v. Romeo, the U.S. Supreme Court tackled the issue of rights for individuals with intellectual and developmental disabilities under state care. The case examined the patient’s right to safe confinement conditions, protection from unnecessary bodily restraint, and access to sufficient medical care and habilitation. The Court emphasized that, in deciding what qualifies as ‘reasonable’ care, courts must rely on the expertise and professional judgment of qualified specialists. Soon after, in the same year, in Rennie v. Klein, the Federal District Court of New Jersey acknowledged that decisions by independent psychiatrists regarding forced treatment should be respected, provided they stem from appropriate professional evaluation.

Given the Rogers statute, in Massachusetts, access to antipsychotic medications over objection requires the court to commit an individual to an inpatient treatment setting, which is followed by a psychiatrist’s treatment petition to the court, called a “Rogers Treatment Plan (MGL Ch. 123 §8B).” This treatment petition awaits an adversarial hearing, and the patient remains in a locked setting until the hearing date. As background, the United States is a common-law country and uses the adversarial court system, where two lawyers represent their positions for the plaintiff and respondent in front of an impartial judge and/or jury. The treatment petition undergoes the same adversarial process as other matters would in the criminal courts. It is important to note that treatment cannot be administered if the patient and their counsel contest or dispute the commitment to the hospital. The treatment petition will be put on hold until the commitment status is determined, thereby denying access to care.

Since the Rogers case decision in Massachusetts, multiple studies have illustrated the costs, adverse events, and negative impact of delaying antipsychotic treatment for those suffering from acute illness while awaiting adversarial hearings.^23^ ^,^^24^ The process of arranging and conducting this hearing takes time, often months while healing, stabilization, and recovery for the sickest patients stall. A study by Schouten and Gutheil published in the American Journal of Psychiatry highlighted the human and economic toll of postponing necessary psychiatric treatment to protect the right to refuse medications legally. The study looked at 2216 Rogers Guardianship petitions that the Massachusetts Department of Mental Health had submitted for patient treatment over an 18-month period. The data showed that the court eventually approved 99.1% of them. Meanwhile, a burdensome and expensive process resulted in significant delays in administering this treatment to patients.^25^ A study published in 2023 on the Rogers Guardianship also showed similar results and documented adverse medical events.

Data published from a strict security forensic hospital in Massachusetts revealed, on average, that it took 61 days, and often longer, to treat patients with medication for serious mental illness due to statute and court-related delays in the Rogers Guardianship pathway for forensic patients. Such a delay in care displayed serious consequences for a patient with a severe mental illness and criminal charges. The study showed that emergency restraints, as well as adverse events, like patient-on-patient assaults, staff assaults, self-harm behaviors, and acute psychotic symptoms like paranoia, persecutory delusions, and hallucinations, decreased significantly in forensic patients once they were finally able to get consistent treatment with a psychiatric treatment provider.^2^ Of the guardianship petitions reviewed in Court, 99.2% were approved, thereby rendering the legal process that caused the treatment delays more of an obstacle than protecting patient decision-making autonomy.

Also, Massachusetts does not permit mandated court-ordered treatment or assisted outpatient treatment in the community, relying instead on Rogers Guardianships. In contrast, almost all other states in the United States allow for mandated court-ordered treatment, particularly for individuals connected to the criminal justice system. However, despite such laws, there is a significant shortage of clinical facilities that provide the necessary specialized services. This shortfall makes it challenging to meet the demand for care among these underserved populations.^26^

## Timely psychiatric treatment improves healthcare equity and reduces healthcare staff burnout and demoralization

The postponement of care due to the Rogers Guardianship process illustrates the high price of solely protecting the autonomy of patients refusing medications who are suffering from severe mental illness.^27^ This cost of delay in treatment not only exacerbates the worsening of the illness by preventing recovery but also increases disparities in healthcare access and increases the cycle of homelessness and incarceration among those who are most vulnerable and least able to bear such burdens. Studies show that mentally ill individuals are much more likely to interact with law enforcement.^28^ ^,^^29^ Multiple factors contribute to the involvement of individuals with mental illness in the criminal justice system.^30^ However, a significant factor is that their active illness is often under-treated or not treated at all, effectively criminalizing mental illness.^31^ ^–^^33^

Equitable care means being fair, inclusive, and timely around access while providing necessary care to patients. While it has been established that timely treatment brings important lifesaving benefits to the individual patient and allows them to live more independently in the community rather than in locked settings, it also promotes equity in under-resourced communities where larger populations of racial diversity stand to gain the most.^34^ ^–^^36^ Certainly, arrest and incarceration should not be the entry point to long-term psychiatric care. Yet, if barriers to accessing care persist, the ongoing trend of doubly stigmatizing the mentally ill through criminal involvement will continue.^37^ Criminalizing mental illness represents a subtle but serious human rights violation in the care of the mentally ill, especially when intensive psychiatric interventions, which the WHO QualityRights initiative currently labels as abuse, are too challenging to implement effectively and in a standard and well-resourced manner.

Timely psychiatric treatment for SMI in the community and shorter bursts in psychiatric units reduce the cycle of homelessness, crime, and incarceration, as well as the negative impacts of long-term institutionalization among those with chronically undertreated psychosis. Treatment in a monitored setting is also important, as these medications require psychiatric expertise to administer, and there should be options to titrate up, taper down, and change medications as the person improves or develops side effects. Proper and adequate treatment can only happen when patients are supported with wraparound services and healthcare staff are supported with resources and feel safe physically, mentally, and legally providing care.

The United States Surgeon General has identified healthcare provider burnout to be on the brink in the 21st century, and in May 2022, he declared the healthcare worker burnout crisis a national priority and called the nation’s stakeholders to action. He cited workplace violence as one major factor contributing to the problem.^38^ In 2019, the WHO defined burnout as an “occupational phenomenon” in the International Classification of Disease (ICD-11)^39^ and noted that up to 38% of healthcare workers have experienced workplace violence and experienced burnout due to these experiences.^40^ A New England Journal of Medicine review article reported that workplace violence against healthcare workers leads to missed workdays, general dissatisfaction, reduced productivity, and burnout. The review identified psychiatric inpatient settings as having the highest risk of workplace violence. It also found that the most common perpetrators of workplace violence are individuals with altered mental status or decompensated mental illness.^41^

Inpatient units, where much of the involuntary treatments take place, often involve a concentration of untreated patients, which may create a hazardous working condition for staff.^41^ ^,^^42^ A clinician’s ability to provide compassionate care is closely tied to feeling safe at their job. When staff feel unsafe, the quality of care they can provide can deteriorate. Additionally, healthcare equity becomes compromised, particularly in areas with more scarce healthcare resources, as healthcare workers are even more susceptible to burnout. Research indicates that burnout in clinical providers can reduce the improvements seen in patients. Timely treatment and reduced barriers to care to accelerate recovery for patients with acute mental illness not only grant them access to equitable and higher quality care but also enhance working conditions and mitigate burnout among healthcare staff.^43^

## Conclusion

There needs to be a balanced approach to the consideration of administering psychiatric interventions involuntarily to deliver life-saving and dignity-restoring care to patients with SMI who need it. While there are many mental healthcare inequities and abuses that do occur globally and should be addressed with sound policies, labeling intensive psychiatric interventions as abusive and violent is problematic. As illustrated by the Rogers Guardianship case example, when psychiatric treatment is postponed or withheld to provide for prolonged legal maneuvering, we risk overlooking the core objectives of mental healthcare. While every effort should be made within a short period of time to determine the capacity for medical decision-making in an individual, this pursuit should not irreversibly deteriorate the patient’s condition in the process of waiting. Prompt treatment not only helps to restore a patient’s decision-making capacity and their ability to live in less restrictive settings, but it also allows them to live with their dignity intact.^7^ Additionally, timely treatment enhances the common goal for healthcare equity and boosts healthcare staff morale, well-being, and a professional clinician’s capacity to deliver compassionate and safe care.

In conclusion, it is paramount that national and global policy acknowledge the need for essential psychiatric care in serious mental illness and actively destigmatize its utilization. Without medication, certain mental illnesses have little chance of recovery. Psychiatric physicians and other mental health professionals find no joy in using involuntary methods in care, yet they ethically weigh this decision against graver consequences. As discussed, these consequences include prolonged and refractory illness, confinement in locked and restrictive settings, diminished access to care for the individuals and those around them, heightened risk of homelessness and incarceration, and the demoralization of healthcare staff and caregivers. The ethical imperative is clear: destigmatizing psychiatric treatment is essential within global policy frameworks if we are to restore the dignity of those living with mental illness.
